# Clinical characteristics of patients with chronic eosinophilic pneumonia in a Chinese tertiary‐care hospital: A 6‐year retrospective study

**DOI:** 10.1111/crj.13448

**Published:** 2021-10-05

**Authors:** Yang Xu, Zhanbo Wang, Wenchao Li, Qiang Zhu, Zhixin Liang

**Affiliations:** ^1^ Department of Respiratory and Critical Care Medicine Chinese PLA General Hospital Beijing China; ^2^ Department of Pathology, the first medical center Chinese PLA General Hospital Beijing China; ^3^ Department of Pediatric Surgery Chinese PLA General Hospital Beijing China

**Keywords:** allergy, chronic eosinophilic pneumonia, clinical manifestations, pathology, treatment

## Abstract

**Introduction:**

Chronic eosinophilic pneumonia (CEP) is a rare disease with unknown etiology. Due to lack of specificity of CEP symptoms, clinicians are not experienced in establishing its diagnosis.

**Objectives:**

To summarize the clinical data of CEP patients to improve the understanding of CEP and reduce misdiagnosis.

**Methods:**

Data of patients pathologically diagnosed with CEP in the PLA General Hospital between May 2013 and May 2019 were collected, and clinical manifestations, imaging characteristics, pathological features, and treatment were retrospectively analyzed.

**Results:**

Twenty patients, including 6 males and 14 females, were diagnosed with CEP. The average age was 47.0 ± 10.2 years. The main clinical manifestations were cough and dyspnea. The average duration of CEP was 15.5 ± 11.5 months. The average proportion of eosinophils in the peripheral blood was 18.9 ± 17.8%, and the average proportion of eosinophils in the bronchoalveolar lavage fluid was 41.5 ± 19.4%. The main imaging features were patchy shadows and consolidation shadows. The most common manifestations on bronchoscopic examination were congestion and edema of the bronchial mucosa. Two patients had granular protrusions of the endotracheal membrane. Histological examination indicated infiltration of numerous eosinophils. All patients improved after prednisone therapy.

**Conclusion:**

CEP onset is insidious, and clinical manifestations lack specificity. Typical imaging features are peripheral and subpleural distribution of lung infiltrates. Some patients have a normal proportion of eosinophils in the peripheral blood, but most have an increased number of eosinophils in the BALF, which contributes to CEP diagnosis. A biopsy is necessary when differential diagnosis is difficult. A systemic glucocorticoid is effective.

## 1 INTRODUCTION

Eosinophilic lung diseases (ELDs) are a group of diseases characterized by varying degrees of pulmonary eosinophilic infiltration or increased peripheral blood eosinophils.^1^ ELDs can be classified into three categories according to the presence or absence of clear pathogenic causes and vascular lesions.^2^ The first category is ELDs with unknown pathogenic causes, including simple pulmonary eosinophilia, acute eosinophilic pneumonia, chronic eosinophilic pneumonia, and idiopathic hypereosinophilic syndrome. The second category is ELDs with clear pathogenic causes, including parasitic infection, drug allergy, and allergic bronchopulmonary aspergillosis. The third category is ELDs with vascular lesions, including allergic vasculitis and allergic eosinophilic granulomatous vasculitis.

Chronic eosinophilic pneumonia (CEP) is a type of ELD that was first reported by Carrington in 1969.^3^ CEP is a rare disease with unknown etiology that has been mostly reported in the form of individual case reports, and there are few systematic studies on this disease entity. Due to the lack of specificity of the signs, symptoms, and imaging manifestations of CEP, clinicians are not experienced in establishing the diagnosis and treatment plan of CEP; thus, missed diagnosis and misdiagnosis are likely. Therefore, we aimed to summarize the clinical data, treatment, and prognosis of patients pathologically diagnosed with CEP in the Respiratory Department of PLA General Hospital to improve the understanding of this disease entity.

## 2 METHODS

### 2.1 Setting

This study was performed at the Chinese PLA General Hospital, a tertiary‐care hospital in Beijing with more than 3400 beds and 200 thousand inpatients per year. This hospital provides medical service to the public.

### 2.2 Patients and design

Twenty patients who were pathologically diagnosed with CEP between May 2013 and May 2019 in the Department of Respiratory and Critical Care Medicine of PLA General Hospital were included in this study. The clinical data of the patients with CEP were collected, analyzed, and summarized; these data included general information, clinical symptoms and signs, and the results of laboratory tests, imaging examinations, pulmonary function examinations, bronchoscopy, cytological classification of the bronchoalveolar lavage fluid (BALF), and pathological examinations as well as the details of treatment and prognosis. All records were independently checked twice by two researchers to avoid any errors.

### 2.3 Statistical analysis

SPSS software, version 20.0 (IBM Corp, Armonk, NY, USA), was used for statistical analysis. The data conformed to a normal distribution, and they are presented as the mean ± standard deviation.

## 3 RESULTS

### 3.1 General conditions

A total of 20 patients, including 6 males and 14 females, with a male‐to‐female ratio of 1:2.3, who were pathologically diagnosed with CEP in the Department of Respiratory and Critical Care Medicine of PLA General Hospital between May 2013 and May 2019 were included in the study. The patient age range at the time of diagnosis was 22–83 years, with an average age of 47.0 ± 10.2 years. The duration of CEP varied from 2 months to 6 years, and the average duration was 15.5 ± 11.5 months. Ten patients had a history of allergic diseases; among them, 6 had a history of bronchial asthma before CEP diagnosis, 2 had a history of allergic rhinitis, and 2 had the history of both bronchial asthma and allergic rhinitis. With respect to misdiagnosis, among the 20 cases, 10 cases were misdiagnosed as pulmonary infection, 4 cases as pulmonary tuberculosis, 4 cases as bronchial asthma, and 2 cases as pulmonary tumors (Table 1).

**TABLE 1:** Demographic and clinical characteristics of patients with CEP

Variable	CEP patients (*n* = 20)
Age (yrs), mean ± SD (range)	47.0 ± 10.2 (22–83)
Gender	
Male	6 (30%)
Female	14 (70%)
Course (mo), mean ± SD (range)	15.5 ± 11.5 (2–72)
Allergic diseases	
Bronchial asthma	6
Allergic rhinitis	2
Bronchial asthma + Allergic rhinitis	2
Misdiagnosed diseases	
Pulmonary infection	10
Pulmonary tuberculosis	4
Bronchial asthma	4
Pulmonary tumors	2
Symptoms	
Cough	18 (90%)
Dyspnea	18 (90%)
Expectoration	10 (50%)
Shortness of breath	9 (45%)
Chest pain	2 (10%)
Hemoptysis	1 (5%)
Fever	1 (5%)
Weight loss	4 (20%)
Asthenia	4 (20%)
Skin urticaria	2 (10%)
Night sweats	1 (5%)
Nausea	1 (5%)
Signs	
Moist rales and scattered wheezes	12 (60%)
Lymphadenectasis	1 (5%)
Hepatosplenomegaly	1 (5%)

### 3.2 Clinical manifestations

The clinical symptoms of 20 patients with CEP lacked specificity, and the common symptoms included cough (18/20 cases), dyspnea (18/20 cases), expectoration (10/20 cases), shortness of breath (9/20 cases), chest pain (2/20 cases), hemoptysis (1/20 cases), fever (10/20 cases), weight loss (4/20 cases), and asthenia (4/20 cases). Individual patients had night sweats, dry mouth and eyes, skin urticaria, nausea, and other manifestations. More than half of the patients (12/20 cases) had wheezing and moist rales on auscultation of the lungs, while extrapulmonary signs, such as lymphadenectasis and hepatosplenomegaly were uncommon (Table 1).

### 3.3 Laboratory test results

The average proportion of eosinophils in the peripheral blood was 18.9 ± 17.8%, and the average peripheral blood eosinophil count was 1.6 ± 3.3 × 10^9^/L. Twelve patients (60%, 12/20 cases) had an elevated peripheral blood eosinophil count, with the proportion of eosinophils ranging from 5.3% to 64.7%, and the absolute value of eosinophils ranged from 0.7 × 10^9^/L to 14.4 × 10^9^/L. Among these 12 patients, 8 patients (66.7%, 8/12 cases) had mild elevation of the peripheral blood eosinophil count, 2 patients (16.7%, 2/12 cases) had moderate elevation of the peripheral blood eosinophil count, and 2 patients (16.7%, 2/12 cases) had severe elevation of the peripheral blood eosinophil count. The average C‐reactive protein (CRP) level was 1.0 ± 0.9 mg/dl, and 9 patients (45%, 9/20 cases) had an elevated CRP level. The average erythrocyte sedimentation rate (ESR) was 29.7 ± 28.0 mm/h, and 10 patients (50%, 10/20 cases) had an elevated ESR. The average IgE level was 254.6 ± 245.5 IU/ml, and 12 patients (60%, 12/20 cases) had an elevated IgE level. There were no significant abnormalities on the (1, 3)‐β‐D glucan test (G test) and galactomannan antigen detection (GM test), tuberculosis‐related examinations, and tumor‐related examinations for all patients (Table 2).

**TABLE 2:** Laboratory, blood gas analysis, and pulmonary function test results for CEP patients

Variable	CEP patients (*n* = 20)
Peripheral blood eosinophil count (×10^9^/L), mean ± SD (range)	1.6 ± 3.3 (0.1–14.4)
Proportion of peripheral blood eosinophils (%), mean ± SD (range)	18.9 ± 17.8 (3.6–64.7)
C‐reactive protein concentration (mg/dl), mean ± SD (range)	1.0 ± 0.9 (0.2–3.9)
Erythrocyte sedimentation rate (mm/h), mean ± SD (range)	29.7 ± 28.0 (2–90)
IgE level (IU/ml), mean ± SD (range)	254.6 ± 245.5 (10–870)
Proportion of eosinophils in the BALF (%), mean ± SD (range)	41.5 ± 19.4 (0–67)
PaO_2_ level (mmHg), mean ± SD (range)	78.9 ± 11.0 (60–96)
Pulmonary function	
Restrictive ventilatory dysfunction	7 (35%)
Obstructive ventilatory dysfunction	5 (25%)
Mixed ventilatory dysfunction	6 (30%)
Normal	2 (10%)

### 3.4 Arterial blood gas and pulmonary function examination

All 20 patients underwent arterial blood gas analysis, and the average partial pressure of oxygen (PaO_2_) was 78.9 ± 11.0 mmHg. All 20 patients underwent pulmonary function tests, which identified restrictive ventilatory dysfunction in 7 patients, obstructive ventilatory dysfunction in 5 patients, mixed ventilatory dysfunction in 6 patients, and no obvious abnormalities in pulmonary function in 2 patients (Table 2).

### 3.5 Imaging findings

**FIGURE 1:**
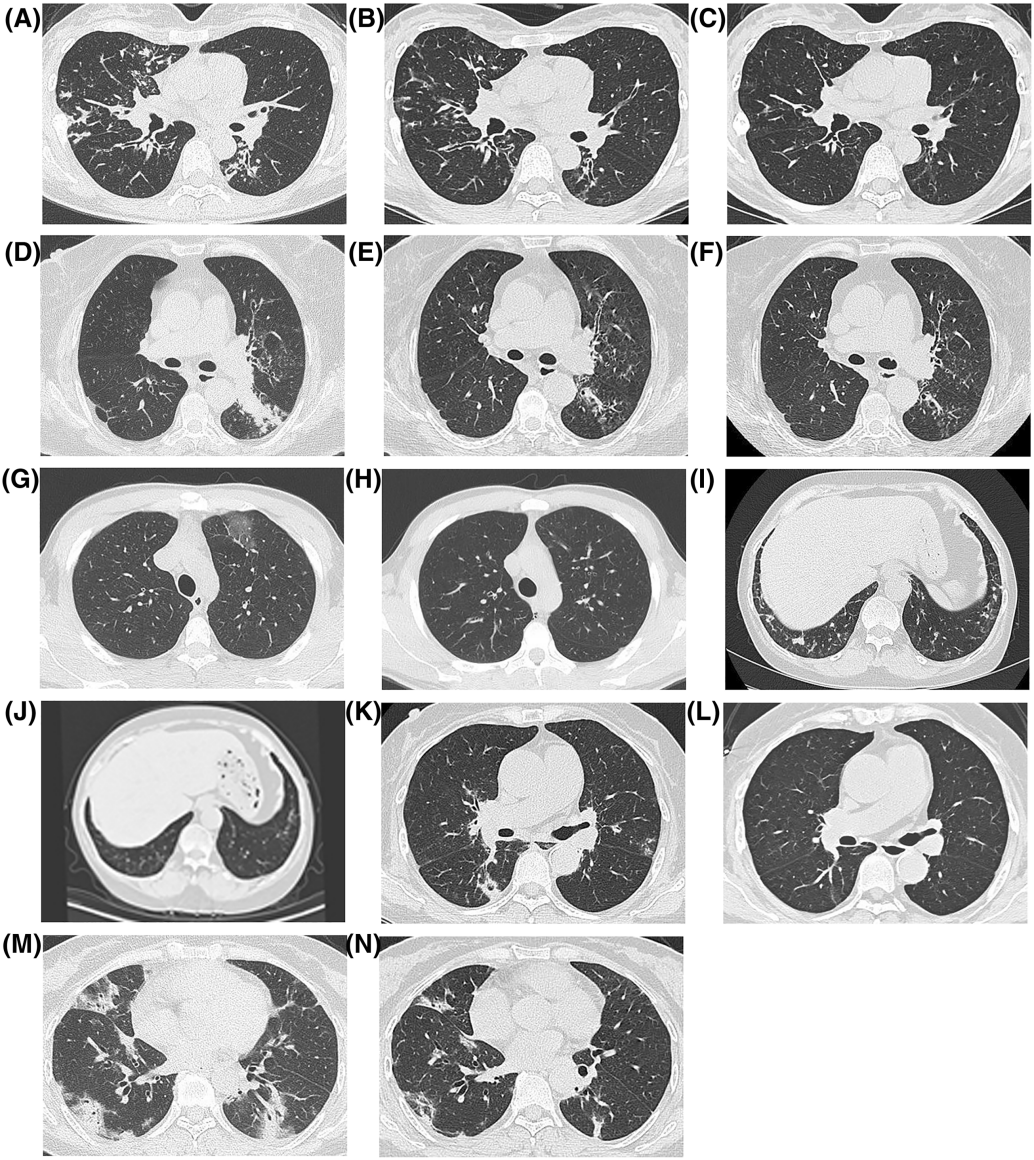
Pulmonary CT of chronic eosinophilic pneumonia. (A) In case 1, pulmonary CT showed scattered patchy shadows in both lungs. (B) After 1 week of glucocorticoid therapy, repeated CT showed that the patchy shadows were reduced. (C) After 1 month of glucocorticoid therapy, repeated CT showed that the patchy shadows had largely disappeared. (D) In case 2, pulmonary CT showed local pulmonary consolidation in the left lower lobe. (E) After 1 week of glucocorticoid therapy, repeated CT showed that the consolidation was reduced. (F) After 1 month of glucocorticoid therapy, repeated CT showed that the consolidation was further reduced. (G) In case 3, pulmonary CT showed ground glass opacities in the left upper lobe. (H) After 1 month of glucocorticoid therapy, repeated CT showed that the ground glass opacities were completely absorbed. (I) In case 4, pulmonary CT showed scattered nodular shadows in both lower lungs. (J) After 1 month of glucocorticoid therapy, repeated CT showed that the nodular shadows had largely disappeared. (K) In case 5, pulmonary CT showed a hollow lesion in the dorsal segment of the lower lobe of the right lung. (L) After 1 month of glucocorticoid therapy, repeated CT showed that the hollow lesion was absorbed. (M) In case 6, pulmonary CT showed multiple patches and solid changes in both lungs. The lesions were mainly distributed under the subpleura and periphery of the lung. (N) After 1 month of glucocorticoid therapy, repeated CT showed that lesions in both lungs were reduced

Chest computed tomography (CT) was performed in all 20 patients. Four patients had unilateral distribution of pulmonary lesions, and 16 patients had bilateral distribution of pulmonary lesions. The lesions were mainly distributed in the periphery or subpleura (Figure 1M,N). The main lesions were patchy shadows (Figure 1A) in 12 cases, consolidation shadows (Figure 1D) in 6 cases, and ground glass shadows (Figure 1G) in 2 cases. Some patients had atypical lesions, including one case with nodular shadows (Figure 1I), one case with cavitary changes (Figure 1K), and one case with pleural effusion.

### 3.6 Bronchoscopy and bronchoalveolar lavage

**FIGURE 2:**
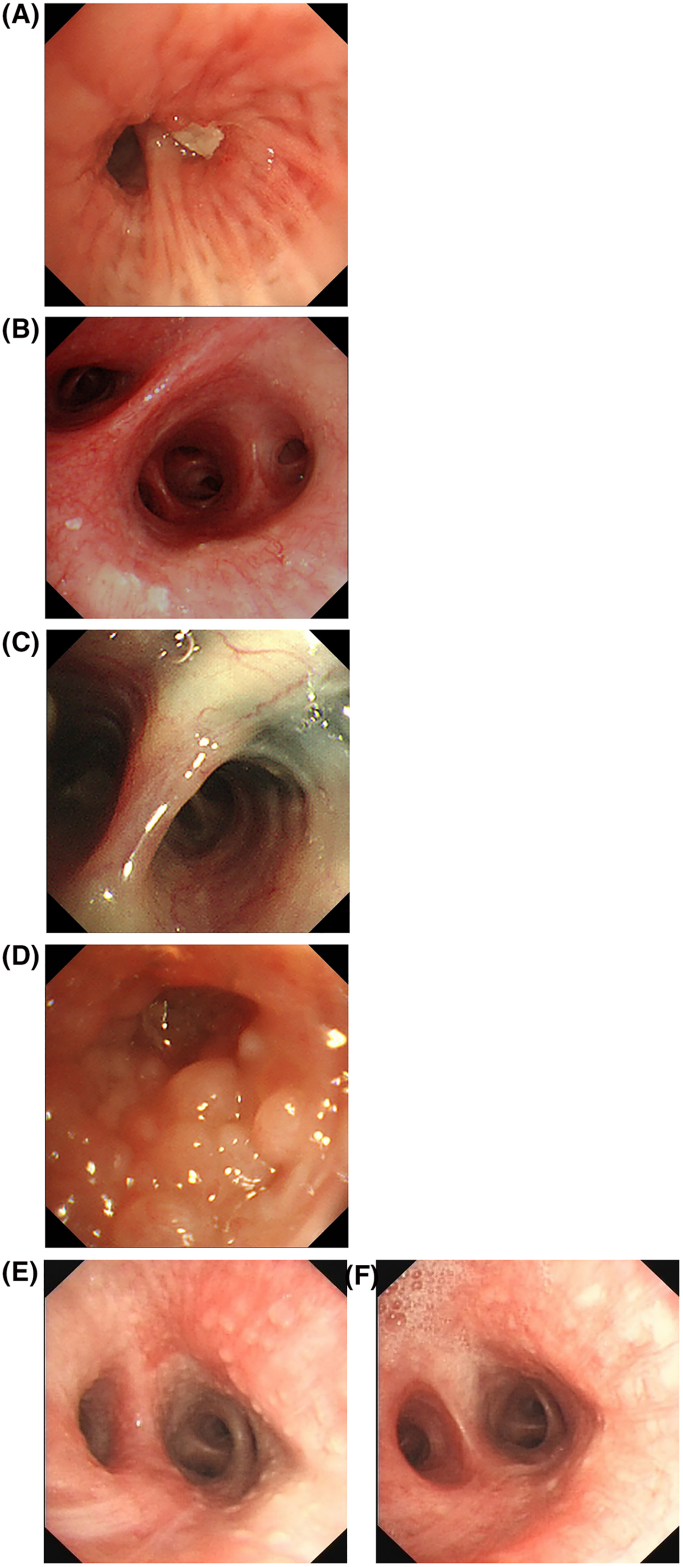
Bronchoscopy of chronic eosinophilic pneumonia. (A) Bronchoscopy revealed edema of the bronchial mucosa in the posterior segment of the upper lobe of the right lung and obstruction of the lumen with white necrosis. (B) Bronchoscopy revealed hyperemia of the bronchial mucosa in the right middle segment, with scattered white membranous secretions on the mucosal surface. (C) Bronchoscopy revealed chronic inflammatory changes locally in the bronchial mucosa with multiple carbon deposition. (D) Bronchoscopy revealed congestion and edema of the bronchial mucosa in the dorsal segment of the left lower lobe with diffuse nodular protrusions on the mucosal surface. (E) Bronchoscopy revealed diffuse granular protrusions on the mucosal surface of the left lower lobe bronchus. (F) Repeated bronchoscopy of the case shown in panel E after 1 month of glucocorticoid therapy revealed a marked decrease in diffuse granular protrusions

All 20 patients underwent bronchoscopy, which showed congestion and edema of the bronchial mucosa in 8 cases (Figure 2A,B), local chronic inflammatory changes in 2 cases (Figure 2C), granular or nodular protrusion of the endotracheal membrane in 2 cases (Figure 2D–F), and no obvious abnormalities in 8 cases. All 20 patients underwent bronchoalveolar lavage, and the average proportion of eosinophils in the BALF was 41.5 ± 19.4%. Further, 18 patients had an increased proportion of eosinophils in the BALF, and the proportion ranged from 12% to 67% (Table 2).

### 3.7 Pathological examination

**FIGURE 3:**
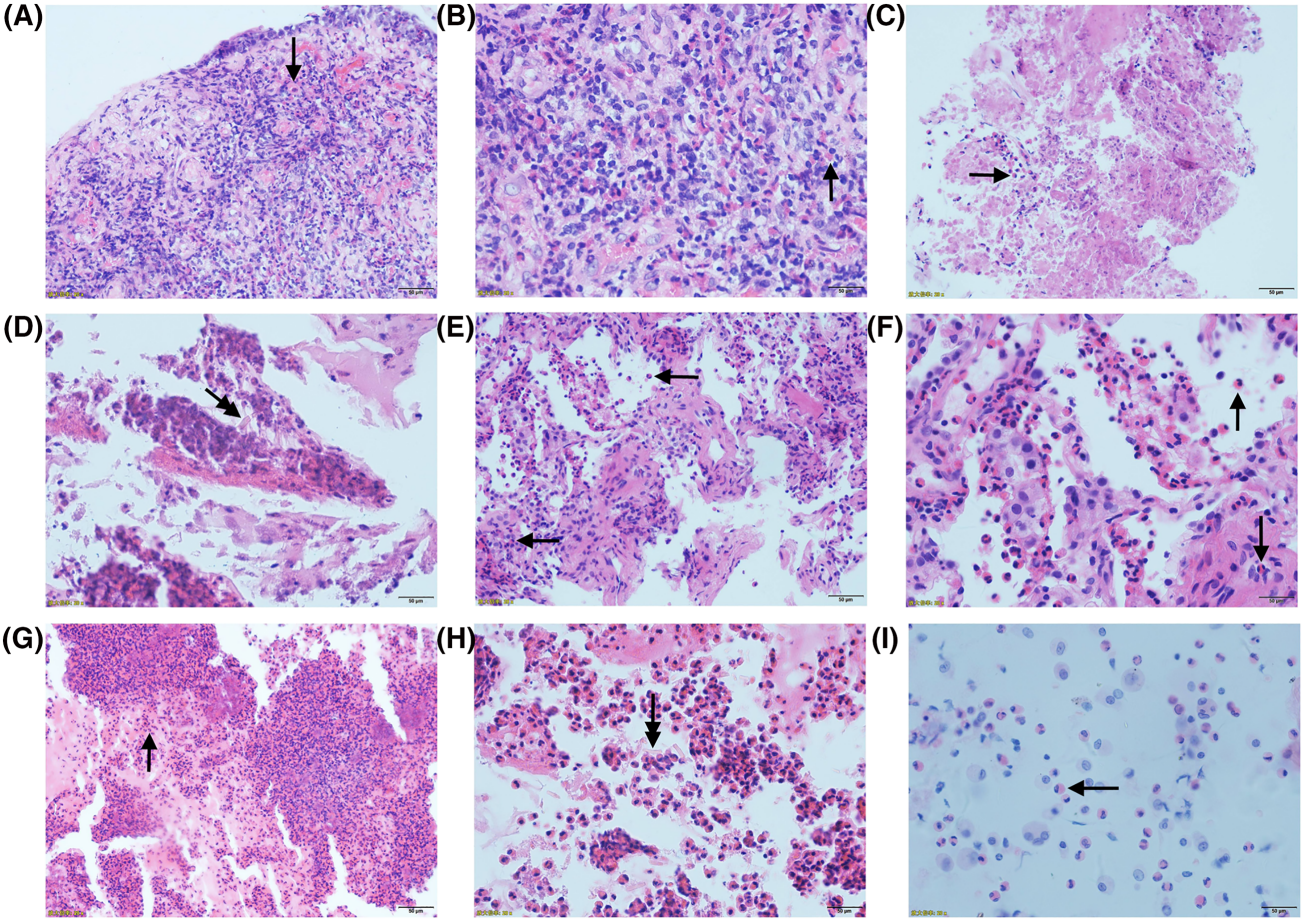
Pathology of bronchoscopic biopsy and pulmonary biopsy of chronic eosinophilic pneumonia. (A, B) Bronchoscopic pathological examination revealed chronic inflammation of respiratory epithelial mucosa with acute inflammation and granulation formation, and edema of the lamina propria with massive eosinophil infiltration (magnification, ×100 in A, ×200 in B). (C, D) Bronchoscopic biopsy revealed eosinophilic abscess formation with localized Charcot‐Leyden crystals (magnification, ×100 in C, ×200 in D). (E, F) Pulmonary biopsy showed local interstitial fibrous proliferation and acute and chronic inflammatory cell infiltration, and a large number of eosinophils infiltration in alveolar cavity and interstitium (magnification, ×100 in E, ×200 in F). (G, H) Pulmonary biopsy revealed eosinophilic abscess formation with localized Charcot‐Leyden crystals (magnification, ×100 in G, ×200 in H). (I) Cytological examination of pleural effusion fluid suggested a marked increase in eosinophils, up to 30% (magnification, ×400). Hematoxylin–eosin staining. Arrows indicate eosinophils, and double arrows indicate Charcot‐Leyden crystals

CEP was confirmed by pathological examination in all 20 cases. Twelve cases underwent bronchoscopic biopsy. Among them, 10 cases demonstrated chronic bronchial mucosal inflammation with massive eosinophil infiltration (Figure 3A,B), some cases showed eosinophilic abscess formation (Figure 3C,D), and some cases showed Charcot‐Leyden crystals (Figure 3D). Percutaneous lung biopsy was further performed in the other 10 cases, including 8 cases with no obvious abnormality under endoscopy and 2 cases with chronic mucosal inflammation on endoscopic biopsy. The histological examination of these 10 cases revealed various degrees of chronic inflammatory cell infiltration in the alveolar cavity and interstitium, and obvious eosinophil infiltration was observed (Figure 3E,F). In addition, eosinophilic abscess formation (Figure 3G,H) and Charcot‐Leyden crystals were identified in some cases (Figure 3H). Moreover, one patient with pleural effusion underwent puncture and drainage of pleural effusion fluid at the same time, and fluid cytology examination showed infiltration of a large number of eosinophils (Figure 3I).

### 3.8 Treatment and follow‐up

Glucocorticoid therapy was recommended, and the starting dose of prednisone was 1.0 mg/kg/d in all 20 patients. Intravenous methylprednisolone (dose equivalent to prednisone 1.0 mg/kg/d) was administered for 1–2 weeks after the diagnosis. After re‐examination of pulmonary imaging for improvement, oral methylprednisolone tablets (dose equivalent to prednisone 0.5 mg/kg/d) were given, with reduction of one tablet every 2 weeks. When the dose of hormone therapy was reduced to the maintenance dose (equivalent to 15 mg of prednisone), the dose was reduced by one tablet every month, and the total course of treatment was more than 6 months. In 17 patients (85%, 17/20 cases), the symptoms improved after 2–3 days of hormone treatment. In 16 patients (80%, 16/20 cases), the proportion of eosinophils in the peripheral blood returned to the normal level after 3–5 days of hormone treatment; and chest imaging improved in 14 patients (70%, 14/20 cases) after 1 week of treatment (Figure 1B,E). In addition, the symptoms of all 20 patients (100%, 20/20 cases) improved after 2 weeks of hormone therapy, and chest imaging improved significantly within 4 weeks (Figure 1C,F,H,J,L,N). After 1–2 years of follow‐up, 19 patients (95%, 19/20 cases) had no relapse, and one patient discontinued the medication without following the medical advice and was re‐admitted later. Hormone therapy was administered again, and the patient's condition improved. Thereafter, the patient insisted on receiving treatment for 6 months, and no recurrence occurred over 2 years of follow‐up.

## 4 DISCUSSION

CEP is a type of ELD of unknown etiology that was first reported by Carrington in 1969.^3^ The epidemiological data indicate that CEP is a rare disease with an incidence of <1/100 000,^4^ and it is mostly reported in the form of individual case reports, with few systematic studies reported for this disease entity. We summarized the clinical and pathological data of patients pathologically diagnosed with CEP to improve the understanding of this disease entity. In our study, the average age of 20 patients with CEP was 47.0 ± 10.2 years, and the ratio of males to females was 1:2.3, which means that middle‐aged women are the group most susceptible to CEP. The duration of disease ranged from 2 months to 6 years, with an average duration of 15.5 ± 11.5 months, which means that CEP has a subacute or chronic onset. In our study, 50% of patients had a history of allergic diseases, most commonly bronchial asthma, followed by allergic rhinitis and sinusitis; thus, CEP may be closely related to allergic diseases.

Symptoms of CEP lacked specificity, and the main clinical manifestations in this study were cough, dyspnea, expectoration, and shortness of breath, often accompanied by fever, weight loss, and asthenia. CEP is mainly confined to the lung and respiratory tract, but it also has extrapulmonary manifestations, such as pericardial effusion, arthralgia, neuropathy, nonspecific skin manifestations, and abnormal liver function, which can be easily confused with allergic eosinophilic granulomatous vasculitis or idiopathic hypereosinophilic syndrome.^5^, ^6^ The symptoms of CEP are atypical, and this disease entity can be misdiagnosed as pneumonia, tuberculosis, and lung cancer.^7^, ^8^, ^9^ In cases of antibiotic‐refractory “pneumonia” and “asthma” for which inhaled hormones have a poor therapeutic effect, or “tuberculosis” without improvement following anti‐tuberculosis treatment, CEP should be suspected.

It has been reported that an elevated peripheral blood eosinophil count contributes to the diagnosis of CEP.^10^ In our present study, 60% of patients had an elevated peripheral blood eosinophil count, with the proportion of eosinophils ranging from 5.3% to 64.7%. Further, 40% of patients showed no elevation of eosinophil count in peripheral blood; thus, the diagnosis of CEP could not be ruled out in patients with a normal proportion of eosinophils in the peripheral blood. CEP may present with non‐specific anemia or increases in C‐reactive protein level and ESR. An elevated serum IgE level was observed in some cases, suggesting the presence of allergic factors in some patients with CEP.

Hypoxemia can occur in the acute phase of CEP. Among the 20 patients in our study, 10 patients had mild hypoxemia and 2 patients had carbon dioxide retention, which indicated that infiltration of eosinophils in the lung had an impact on the respiratory function of patients. The main changes in pulmonary function in CEP were restrictive ventilatory disorder and diffuse hypofunction. Obstructive ventilatory disorder occurred in patients with asthma, and some patients developed mixed ventilatory dysfunction in the later stage. After treatment, pulmonary function improved rapidly. Recent studies have revealed that reduced lung CO diffusing capacity is a significant predictor of subclinical disease activity and recurrence in patients with CEP.^11^

The lesions on pulmonary imaging of the 20 cases were bilateral, non‐migratory with clear boundaries, and they were mainly distributed in the periphery or subpleura. The most common lesions were patchy shadows, followed by consolidation and ground glass shadows. Some cases had atypical changes, such as nodular shadows, cavity formation, and pleural effusion. The imaging manifestations of CEP are diverse and can be misdiagnosed.

Bronchoscopic examination of the 20 patients showed that congestion and edema of the bronchial mucosa were the most common manifestations. In our study, granular or nodular protrusions were observed in the endotracheal membrane of two patients and the histological examination of bronchial mucosal biopsy indicated eosinophilic infiltration in submucosal tissues. Airway involvement is rare, but it requires attention. In our study, 90% of patients had an increased proportion of eosinophils in the BALF, and the proportion ranged from 12% to 67%. The proportion of eosinophils in the BALF was increased in some patients without obvious abnormal changes under bronchoscopy, which reflected the important value of bronchoscopic alveolar lavage in the diagnosis of CEP. It has been reported that a proportion of eosinophils in the BALF >25% is suggestive of CEP.^12^ In our study, the proportion of eosinophils in the BALF was <25% in some cases, and the pathology of pulmonary puncture indicated CEP; thus, the demarcation line of 25% for the diagnosis of CEP may be stringent.

If the patient is suspected of having CEP and the differential diagnosis is difficult, bronchial mucosal biopsy and pulmonary biopsy are recommended. Histological examination of bronchial mucosal biopsy indicated eosinophilic infiltration in submucosal tissues, and pulmonary biopsy indicated infiltration of a large number of eosinophils in the alveolar cavity and septum, which were the main pathological changes in CEP. Aggregation of eosinophils and necrosis form an ‘eosinophilic abscess’, and Charcot‐Leyden crystals can also be found in some cases. The clinical and imaging manifestations of CEP are complex and diverse, and the diagnosis of CEP can be confirmed by pathology.

Glucocorticoids are the most effective drug for CEP.^13^ There is no unified standard for the dosage and course of glucocorticoid therapy. There is a possibility of recurrence of CEP during the course of hormone reduction or after withdrawal. It has also been reported that when the hormone dose was reduced to 15 mg, the disease could easily recur; thus, the process of hormone dose reduction should be performed with caution. In our study, the initial drug for glucocorticoid treatment in 20 patients was methylprednisolone (dose equivalent to prednisone 1.0 mg/kg/d), which was intravenously administered for 1–2 weeks. After improvement was noted on chest imaging, oral methylprednisolone tablets (dose equivalent to prednisone 0.5 mg/kg/d) were used, and the dose was gradually reduced to a minimum maintenance dose of 15 mg. The total course of treatment was more than 6 months, and the overall therapeutic effect was good.

Suplatast tosilate is an orally active Th2 cytokine inhibitor that can inhibit production of both IL‐4 and IL‐5 by Th2 cells and suppress IgE synthesis. Suplatast tosilate has antiasthmatic, anti‐inflammatory, and antifibrotic activities. In recent years, a few case reports have demonstrated the successful treatment of CEP with suplatast tosilate due to intolerance of hormone therapy.^14^ It has also been reported that monoclonal antibody omalizumab has a significant effect in the treatment of CEP.^15^, ^16^, ^17^ Monoclonal antibody omalizumab is an anti‐IgE antibody used in asthma and other allergic diseases. The therapeutic mechanisms of suplatast tosilate and omalizumab in the treatment of CEP need to be studied further. Previous studies have reported that eosinophils play an important role in the occurrence and development of CEP, and there are targeted drugs for eosinophil recruitment, eosinophil activation, and eosinophil apoptosis induction.^18^, ^19^ However, whether these drugs can be used in the treatment of CEP requires further investigation.

## 5 CONCLUSION

Because CEP is a rare disease, the number of cases included in this article is small. If patients have respiratory symptoms lasting for more than 2 weeks, an increased eosinophil count in the BALF and/or peripheral blood, and chest imaging shows non‐segmental patchy shadows and solid shadows under the subpleura or in the periphery of the lung, the possibility of CEP should be considered. Airway involvement is rare, but it requires attention. Because the clinical and imaging manifestations of CEP are complex and diverse, in cases with difficult diagnoses, CEP can be confirmed by pathology. Glucocorticoid therapy is the most effective treatment for CEP; an initial dosage of prednisone of 1.0 mg/kg/d and a total course longer than 6 months are effective. New monoclonal antibodies against eosinophilic inflammation can also provide a novel direction for the treatment of CEP, and active investigation of the etiology and pathogenesis can provide new ideas for the diagnosis and treatment of CEP.

## CONFLICT OF INTEREST

The authors have no conflicts of interest to declare.

## ETHICS STATEMENT

The local institutional review board (Ethics Committee of PLA General Hospital) approved this study (S2020‐301).

## AUTHOR CONTRIBUTIONS

Y. X. made contributions to analyzing and interpreting the data and was a major contributor in writing the manuscript. Z. B. W. performed the pathological examination. W. C. L. made contribution to acquisition of the imaging data. Q. Z. performed the bronchoscopic examination. Z. X. L. made substantial contributions to conception and design of the study. All authors read and approved the final manuscript.

## Data Availability

The datasets generated and analyzed during the current study are available from the corresponding author on reasonable request.
